# The relationship between *TNF-α* gene promoter polymorphism (− 1211 T > C), the plasma concentration of TNF-α, and risk of oral mucositis and shortening of overall survival in patients subjected to intensity-modulated radiation therapy due to head and neck cancer

**DOI:** 10.1007/s00520-019-04838-6

**Published:** 2019-05-10

**Authors:** Radosław Mlak, Tomasz Powrózek, Anna Brzozowska, Iwona Homa-Mlak, Marcin Mazurek, Paweł Gołębiowski, Grzegorz Sobieszek, Teresa Małecka-Massalska

**Affiliations:** 1 Department of Human Physiology, Medical University of Lublin, Radziwiłłowska 11, 20-080 Lublin, Poland; 2 Department of Oncology, Medical University of Lublin, Lublin, Poland; 3 Clinic of Cardiology and Internal Medicine, Department of Cardiology, Military Hospital, Lublin, Poland

**Keywords:** TNF-α, Polymorphism, Radiotherapy, Head and neck cancer, Oral mucositis, Overall survival

## Abstract

**Purpose:**

Radiotherapy (RTH) usually combined with chemotherapy (C-RTH) is the main method of treatment in head and neck cancer (HNC). The most common complication of RTH is oral mucositis (OM). At a certain stage of RTH, it occurs in almost all patients, often lead to discontinuation of treatment. Tumour necrosis factor alpha (TNF-α) is a cytokine secreted during inflammatory process accompanying RTH and the development of cancer itself. Single nucleotide polymorphism (SNP) of the *TNF-α* promoter region can potentially affect the function or expression of this cytokine, and thus modulate the risk of occurrence and intensity of OM and shortening of overall survival (OS).

**Methods:**

The study group consisted of 62 patients with HNC in whom intensity-modulated radiation therapy (IMRT) technique was applied. The plasma TNF-α level was assessed using the ELISA Kit. Genotyping was performed using a real-time PCR method.

**Results:**

HNC patients with the CC genotype of *TNF-α* (− 1211 T > C) have higher TNF-α plasma concentrations than those with T allele (10.70 vs 9.62 ng/ml). Patients with the 3rd degree of OM have significantly higher TNF-α levels after 5th (10.40 vs 9.45 ng/ml) and 7th (10.32 vs 9.60 ng/ml) week of RTH. CC genotype was related to a higher risk of 3rd degree OM development in the last weeks of RTH (5th, OR = 7.33; 7th, OR = 23.15).

**Conclusions:**

High TNF-α plasma concentration and CC genotype of *TNF-α* are related to the higher risk of more severe OM in patients irradiated due to HNC. High TNF-α plasma concentration and CC genotype of *TNF-α* are independent prognostic factors for patients subjected to RTH due to HNC.

**Electronic supplementary material:**

The online version of this article (10.1007/s00520-019-04838-6) contains supplementary material, which is available to authorized users.

## Introduction

Head and neck cancers (HNC) are the sixth most frequent group of cancers in the world. Over half a million cases are diagnosed each year. HNC form a heterogeneous group of tumours occurring in various anatomic locations, including in the mouth, throat, paranasal sinuses, salivary glands and larynx. The most frequently occurring histological type in this group is squamous cell carcinoma (HNSCC) found in about 90% of cases. Every year, HNSCC is responsible for nearly 2% of deaths due to malignant tumours [1, 2]. Radiotherapy (RTH) usually associated with chemotherapy (C-RTH) is the most frequently used method of therapy in patients with HNC. One of the most common complications of RTH is oral mucositis (OM). OM can develop in every part of the gastrointestinal tract but the most common region for lesions is the mucus of the tongue, cheeks, soft palate and lips [3]. At a certain stage of RTH alone or C-RTH, OM occurs in almost all patients, constituting a serious limitation and often leads to discontinuation of treatment [4]. OM can be described as a gradually increasing oedema of mucous membranes, ulcerations, oral erythema leading to pain and dysphagia. The most common scales used to assess the severity of OM are based on Radiation Therapy Oncology Group/European Organisation for Research and Treatment of Cancer (RTOG/EORTC) scale. Above scale include four grades. Grade 1—mild, oral pain and diffuse erythema occur but the patient can still eat solid food. Grade 2—moderate, erythema and small foci of ulcers are appearing but oral intake is preserved (soft diet). Grade 3—severe, painful ulcers extending to more than half of the oral mucosa, do not allow oral intake but liquids intake are preserved. Grade 4—painful ulcers covering almost all mucosal surfaces, any alimentation by oral is impossible [3, 5, 6]. Serve OM (3rd–4th degree) can be found in nearly 1/3 of patients with HNC [4, 7]. Severe OM may lead not only to worsening of the quality of patients’ life, need for additional hospitalizations but also to interruptions in RTH (approximately 35% HNC patients with severe OM require delay or discontinuation of CTH and in 60% of them a reduction of doses is necessary) [4, 8]. The inability to apply full treatment limits both the possibility of obtaining local disease control and is associated with shorter overall survival (OS), because every 5 days of interruption in RTH increases the risk of progression by nearly 15% [9]. To date, only a few following factors have been found to be related with more severe OM: male gender, older age, poor oral hygiene, a higher dose of radiation, smoking during treatment and introduction of C-RTH [10]. Nevertheless, the observed high individual variation in the degree of OM in irradiated patients indicates the significant role of genetic predispositions (e.g. single nucleotide polymorphisms; SNPs) [11]. The development of OM is caused by the direct influence of ionising radiation on mucosal cells as well as the secretion of pro-inflammatory cytokines, e.g. tumour necrosis factor alpha (TNF-α). TNF-α, released mainly by activated macrophages, is a pleiotropic and pro-inflammatory cytokine. TNF-α is a cytokine secreted as part of the inflammatory process that accompanies RTH and the development of cancer itself. It is responsible for the regulation of two opposite processes: proliferation and apoptosis [12]. The apoptotic pathway is activated by TNF-α through the TNF1 receptor (TNFR1) and probably apoptotic disorders in OM may be caused by the ligand level for this receptor [13]. Single-nucleotide polymorphism (SNP) of the *TNF-α* promoter region can potentially affect the function or expression of this cytokine, and thus modulate the risk of OM development or exacerbation as well as progression and shortening of OS. So far, several studies try to evaluate the association between and specific genetic alterations (SNPs, mutations, expression, miRNA) in the genes encoding proteins (ligands or their receptors) involved in inflammatory processes and thus potentially related with occurrence and severity of OM as well as OS in HNC patients treated with RTH [11, 12]. Therefore, the aim of this study was the evaluation of the relationship between SNP (− 1211 T > C, rs1799964) of *TNF-α* gene as well as TNF-α plasma concentration and the occurrence and intensity of OM and risk of OS shortening in HNC patients subjected to RTH.

## Materials and methods

### Patients and clinical data

We included in our study 62 patients with histologically confirmed, advanced HNC (96.8% of patients were in III or IV stage of disease according to VII edition of TNM classification). All patients were diagnosed between 2014 and 2015 and treated in the Oncology Department in Medical University of Lublin. Inclusion criteria were age at least 18 years old, any gender and had neoplastic lesions in the head and neck region. Moreover, only patients who received a total dose of radiation, treated with intensity-modulated radiation therapy (IMRT) either after surgery or as a definitive treatment modality, with or without sequential and/or concurrent chemotherapy, were included in the study. Exclusion criteria were infection or salivary gland tumours, Sjögren’s syndrome, diagnosis of lymphoma, melanoma or skin cancer or any prior malignancies. Performance status of patients was evaluated according to criteria developed by Eastern Cooperative Oncology Group (ECOG). Alcohol consumption was assessed in accordance with the criteria to the International Statistical Classification of Diseases and Related Health Problems (ICD) as excessive (F 10.1 and F 10.2) or occasional. Current smoker: an adult who has smoked 100 cigarettes in his or her lifetime and who currently smokes cigarettes. Former smoker: person described above who had quit smoking at the time of interview. Smoker: former or current smoker. Never smoker: an adult who have never smoked, or who has smoked less than 100 cigarettes in his or her lifetime. OM presence was evaluated with the use of RTOG/EORTC scale before the start of the treatment and after every week of RTH. Due to the low frequency and considerable diversity of other RTH-induced toxicities, they were not included in the analysis. The OS was calculated in months—from the start of treatment until the end of the observation or death. Baseline characteristic of the study group is presented in Table 1. This project was approved by the Bioethical Commission in Medical University in Lublin (KE-0254/232/2014). Patients were informed about the purpose of the study and they signed consent for this research.

**Table 1:** Demographic and clinical characteristics of study group

Factor		Study group (*n* = 62)
Gender	Male	51 (82.2%)
	Female	11 (17.8%)
Age, median (range)		63 (42–87)
	≥ 63	32 (48.4%
	< 63	30 (51.6%)
Histopathological diagnosis	Squamous cell carcinoma	57 (91.9%)
	Other	5 (8.1%)
Tumour location	Upper throat	17 (27.4%)
	Lower throat	45 (72.6%)
	Larynx	34 (54.8%)
	Others	28 (45.2%)
T stage	T1	2 (3.2%)
	T2	9 (14.5%)
	T3	15 (24.2%)
	T4	36 (58.1%)
N stage	Nx	2 (3.2%)
	N0	18 (29%)
	N1	6 (9.7%)
	N2	32(51.6%)
	N3	4 (6.5%)
M stage	Mx	3(75%)
	M1	1 (25%)
Disease stage	I	2 (3.2%)
	III	12 (19.4%)
	IVA	40 (64.5%)
	IVB	3 (4.8%)
	IVC	5 (8.1%)
Performance status (PS)	≤ 1	55 (88.7%)
	> 1	7 (11.3%)
Type of treatment	Neoadjuvant chemotherapy	10 (16.1%)
	Prior surgical treatment	44 (71%)
	Concurrent chemotherapy	24 (38.7%)
Alcohol consumption	Yes	28 (45.2%)
	No	34 (54.8%)
Smoking status	Smoker	52 (83.9%)
	Non-smoker	10 (16.1%)
	Current smoker	45 (86.5%)
	Former smoker	7 (13.5%)

### Radiotherapy

For radical RTH, linear accelerator ONCOR (Siemens) was used. In all patients, IMRT technique with total doses of 54–70 Gy (daily dose 2 Gy) was applied. Patients with the gross disease were treated with a total dose 70 Gy in 35 fractions for tumour and enlarged lymph nodes. Doses of 54 Gy or 60 Gy were used in the treatment of elective lymph nodes. Patients after surgical resection were given a dose of 66 Gy in 33 fractions for high-risk volume; the intermediate and low-risk subclinical volumes received 60 Gy and 54 Gy, respectively. Moreover, some patients were treated with chemotherapy (concurrent or neoadjuvant) based on cisplatin and 5-fluorouracil (PF) schemes. One to four courses of chemotherapy was administered.

### Genotyping/ELISA testing

From all study participants, 5 ml of whole blood was obtained (stored in − 80 °C). DNA Blood Mini Kit (Qiagen, Canada) was used to DNA isolation. DNA quality and quantity were evaluated with the use of NanoDrop Lite Spectrophotometer (Thermo Fisher Scientific, USA). Genotyping was performed using a real-time PCR method with allele-discriminating software. The Genotyping Master Mix and TaqMan probes (Applied Biosystems, USA) specific for studied *TNF-α* SNP (Thermo Fisher Scientific, USA, SNP ID: C_7514871_10, cat. no. 4351379) were used to DNA amplification under the condition of manufacturer protocol provided with kit in RT7500 Real-time PCR device (Applied Biosystems, USA). Because we were interested in the assessment of the generalised inflammation process (TNF-α in circulation) instead of local changes (TNF-α in saliva), we decided to use plasma instead of saliva as a test material. The plasma TNF-α level was assessed using TNF alpha Human ELISA Kit Ultrasensitive (Thermo Fisher Scientific, USA, cat. no. KHC3014). The detection range was 0.2–32 pg/mL, and the sensitivity was equal to the minimal detectable dose of this kit (< 0.09 pg/mL).

### Statistical analysis

Statistical analysis was conducted using MedCalc version 12.7 software (MedCalc Software, Belgium). We assessed sample size retrospectively, and the results were extrapolated to the input section of the post-hoc test chart. We followed the calculation by using criteria: study group—independent (one vs population) and primary endpoint as continuous. Because of the retrospective manner of post-hoc, we analysed statistically significant results. The post-hoc parameters also included anticipated means and we defined the errors types (type I and type II error). Most of the studies considered *p* value below 0.05 to reject the null hypothesis; the type I error (alpha) of 0.05 value was used. As for type II error (beta), most medical literature uses a beta cut-off of 20% (0.2)—indicating a 20% chance that a significant difference is missed; therefore, type II error was set on 0.2. Considering the prevalence of severe OM in the study group (41.9%) and the general population (60%), the minimal group of patients to deal with a study hypothesis was calculated and estimated to 58 samples. The Fisher’s exact test and Chi-squared test were used to evaluation of the balance of Hardy-Weinberg equilibrium and to compare the distribution of demographic and clinical factors among patients with different genotypes of the *TNF-α* gene. Differences in TNF-α concentration (continuous variable) among patients with different TNF-α genotypes and OM status were analysed by U Mann-Whitney rank sum test and ANOVA Kruskal-Wallis test. Receiver operating curves (ROC) with an area under the curve (AUC) were generated to predict the development of grade 3 of OM among studied patients. Odds ratio (OR) with 95% confidence interval (95% CI) test was applied to assess OM risk related to studied demographic and clinical factors. Kaplan-Meier estimator and Cox regression model were applied to assess factors (with hazard ratio calculation–HR) affecting patients’ survival. Factors with a level over median were considered as high, whereas this below median range was assessed as low. Results with *p* < 0.05 were considered as statistically significant.

## Results

**Fig. 1:**
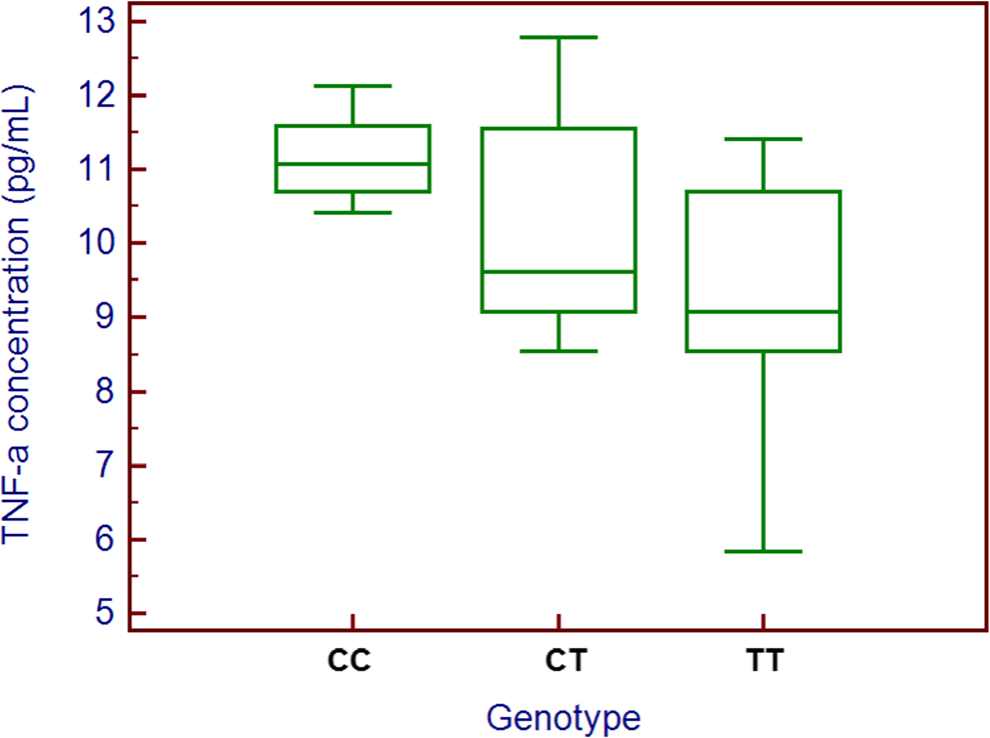
TNF-α plasma concentration according to *TNF-α* genotype

Distribution of *TNF-α* genotypes (CC - 9.7%, CT - 30.6%, TT - 59.7%) was in Hardy-Weinberg equilibrium (*p* = 0.1501; *χ*^2^ = 2.07) and was not significantly different among patients divided based on demographic and clinical factors (supplementary Table 1). CC genotype carriers had significantly higher TNF-α plasma concentrations compared to patients with other SNP variants (10.70 vs 9.62 ng/ml, *p* = 0.008). Moreover, patients with TT genotype had lower TNF-α plasma concentrations, than those with C allele (9.08 vs 9.98, *p* = 0.0015) (supplementary Table 2) (Fig. 1). During subsequent RTH weeks, every patient developed OM (grades 0–3). With each week of RTH—intensification of OM reaction was observed. Starting from the 2nd week of RTH, all patients developed OM (to varying degrees). However, in the first 2 weeks, there were no cases with 3 grade of OM. In the 3rd week, its frequency was equal to 11.3%. After the finish of RTH (in the 7th week), 3rd degree of OM has been found in over 40% of patients. None of the studied demographic and clinical factors influenced the risk of the 2nd or 3rd degree of OM development. Similar results were obtained when the risk of a 3rd degree of OM was assessed. In this case, the only exception was concurrent chemotherapy. Introduction of C-RTH was associated with an approximately sixfold higher risk of developing of the 3rd grade of OM both after the 5th RTH week (OR = 6.07, 95% CI 1.63–22.64, *p* = 0.007) and 7th RTH week (OR = 5.60, 95% CI 1.83–17.07, *p* = 0.002) but not after 6th week (supplementary Tables 3–4). We noted significantly higher TNF-α levels in patients with a 3rd degree of OM compared to those with lower intensive RTH-induced reactions (1st and 2nd degree) after 5th (10.40 vs 9.45 ng/ml, *p* = 0.020) and 7th (10.32 vs 9.60 ng/ml, *p* = 0.043) week of RTH (supplementary Table 5). The presence of CC genotype was related with over sevenfold (OR = 7.33, 95% CI 1.120–44.96, *p* = 0.031) and 23-fold (OR = 23.15, 95% CI 1.24–432.14, *p* = 0.035) higher risk of 3rd degree OM development after the 5th and 7th week of RTH, respectively. Similarly, C allele carriers (CC and CT genotypes) had a significantly higher risk of more serve (3rd grade) OM after the 7th week of RTH (OR = 6.61, 95% CI 2.14–20.40, *p* = 0.001). Influence of studied genotypes and TNF-α level on the occurrence of more serve OM was showed in Table 2 and supplementary Table 5. Considering the TNF-α level, we used ROC analysis to set a concentration cut-off point to diagnose the occurrence of more serve OM after subsequent weeks of RTH in the study group. For the 5th week: the diagnostic accuracy in 3rd grade OM detection was: 61.5% sensitivity and 81.4% specificity (associated criterion > 10.54 ng/ml; AUC = 0.713, 95% CI 0.579–0.824, *p* = 0.018), whereas for the 7th week, it was: 56.5% and 70.6% respectively (associated criterion > 10.08 ng/ml; AUC = 0.653 95% CI 0.515–0.774, *p* = 0.046). All ROC curves analysis results were demonstrated in Table 3.

**Table 2:** Impact of *TNF-α* genotypes on the risk of more severe oral mucositis after subsequent cycles of RTH

RTH cycle	Radiation reaction grade	CC (*n* = 6; %)	TT or CT	*p*, OR [95% CI]	TT (*n* = 37; %)	CC or CT	*p*, OR [95% CI]	CT (*n* = 19; %)	CC or TT	*p*, OR [95%CI]
1	0 (*n* = 6; 9.7%)	–	6 (100%)	0.736 1.673 [0.084–33.281]	5 (83.3%)	1 (16.7%)	0.241 3.750 [0.411–34.230]	1 (16.7%)	5 (83.3%)	0.446 2.368 [0.258–21.787]
	1 (*n* = 56; 90.3%)	6 (10.7%)	50 (89.3%)		32 (57.1%)	24 (42.9%)		18 (32.1%)	38 (67.9%)	
2	1 (*n* = 36; 58%)	2 (5.6%)	34 (94.4%)	0.214 3.091 [0.521–18.331]	23 (63.9%)	13 (36.1%)	0.427 1.517 [0.542–4.240]	11 (30.6%)	25 (69.4%)	0.930 1.051 [0.353–3.128]
	2 (*n* = 26; 42%)	4 (15.4%)	22 (84.6%)		14 (53.8%)	12 (46.2%)		8 (30.8%)	18 (69.2%)	
3	1 (*n* = 21; 33.9%)	1 (4.8%)	20 (95.2%)	0.366 2.778 [0.303–25.463]	14 (66.6%)	7 (33.3%)	0.424 1.565 [0.523–4.689]	6 (28.6%)	15 (71.4%)	0.800 1.161 [0.367–3.676]
	2 and 3 (*n* = 41; 66.1%)	5 (12.2%)	36 (87.8%)		23 (56.1%)	18 (43.9%)		13 (31.7%)	28 (68.3%)	
	1 and 2 (*n* = 55; 88.7%)	5 (%)	50 (%)	0.664 1.667 [0.166–16.758]	35 (63.6%)	20 (36.4%)	0.094 4.374 [0.776–24.664]	15 (27.3%)	40 (72.7%)	0.123 3.556 [0.711–17.793]
	3 (*n* = 7; 11.3%)	1 (9.1%)	6 (90.9%)		2 (28.6%)	5 (71.4%)		4 (57.1%)	3 (42.9%)	
4	1 (*n* = 11; 17.7%)	–	11 (100%)	0.429 3.286 [0.172–62.673]	9 (81.8%)	2 (18.2%)	0.116 3.696 [0.725–18.837]	2 (18.2%)	9 (81.8%)	0.332 2.250 [0.437–11.589]
	2 and 3 (*n* = 51; 82.3%)	6 (11.8%)	45 (88.2%)		28 (64.9%)	23 (45.1%)		17 (33.3%)	34 (66.7%)	
	1 and 2 (*n* = 49; 79%)	4 (8.2%)	45 (91.8%)	0.441 2.045 [0.331–12.636]	32 (65.3%)	17 (34.7%)	0.090 3.012 [0.852–10.647]	13 (26.5%)	36 (73.5%)	0.179 2.374 [0.672–8.380]
	3 (*n* = 13; 21%)	2 (15.4%)	11 (84.6%)		5 (38.5%)	8 (61.5%)		6 (46.2%)	7 (53.8%)	
5	1 (*n* = 6; 9.7%)	–	6 (100%)	0.736 1.673 [0.084–33.281]	6 (100%)	–	0.115 10.524 [0.566–195.81]	–	6 (100%)	0.200 6.760 [0.362–126.36]
	2 and 3 (*n* = 56; 90.3%)	6 (10.7%)	50 (89.3%)		31 (65.4%)	25 (44.6%)		19 (33.9%)	37 (66.1%)	
	1 and 2 (*n* = 48; 77.4%)	4 (8.3%)	44 (91.7%)	0.512 1.833 [0.299–11.241]	31 (64.6%)	17 (35.4%)	0.151 2.431 [0.723–8.175]	13 (27.1%)	35 (72.9%)	0.265 2.019 [0.587–6.944]
	3 (*n* = 14; 22.6%)	2 (14.3%)	12 (85.7%)		6 (42.9%)	8 (57.1%)		6 (42.9%)	8 (57.1%)	
6	1 (*n* = 7; 11.3%)	–	7 (100%)	0.655 1.970 [0.100–38.673]	7 (100%)	–	0.089 12.541 [0.683–230.37]	–	7 (100%)	0.162 8.014 [0.434–147.85]
	2 and 3 (*n* = 55; 88.7%)	6 (10.9%)	49 (89.1%)		30 (54.5%)	25 (45.5%)		19 (34.5%)	36 (65.5%)	
	1 and 2 (*n* = 46; 74.2%)	2 (4.3%)	44 (95.7%)	0.031* 7.333 [1.196–44.965]	28 (60.9%)	18 (39.1%)	0.746 1.210 [0.283–3.827]	16 (34.8%)	30 (65.2%)	0.239 2.311 [0.573–9.319]
	3 (*n* = 16; 25.8%)	4 (25%)	12 (75%)		9 (56.2%)	7 (43.8%)		3 (18.8%)	13 (81.2%)	
**7**	1 (*n* = 8; 12.9%)	–	8 (100%)	0.587 2.278 [0.117–44.294]	8 (100%)	–	0.069 14.695 [0.808–267.35]	–	8 (100%)	0.132 9.338 [0.511–170.60]
	2 and 3 (*n* = 54; 87.1%)	6 (11.1%)	48 (88.9%)		29 (53.7%)	25 (46.3%)		19 (35.2%)	35 (64.8%)	
	1 and 2 (*n* = 36; 58.1%)	–	36 (100%)	0.035* 23.146 [1.240–432.14]	28 (77.8%)	8 (22.2%)	0.001* 6.611 [2.142–20.405]	8 (22.2%)	28 (77.8%)	0.100 2.567 [0.849–7.755]
	3 (*n* = 26; 41.9%)	6 (23.1%)	20 (76.9%)		9 (34.6%)	17 (65.4%)		11 (42.3%)	15 (57.7%)	

*OR* odds ratio, 95% CI 95% confidence interval, *Statistically significant values

**Table 3:** ROC curve analysis in the evaluation of the usefulness of TNF-α plasma concentration measurements in the detection of a higher grade of oral mucositis after subsequent cycles of RTH

RTH cycle	Radiation reaction grade	*n* (%)	Cut-off	Sensitivity	Specificity	AUC [95% CI]	*p*
1	0	6 (9.7%)	7.68	94.3%	33.3%	0.593 [0.457–0.719]	0.514
	1	56 (90.3%)					
2	1	36 (58%)	11.6	20%	100%	0.526 [0.393–0.657]	0.738
	2	26 (42%)					
3	1	21 (33.9%)	9.5	50%	71.4%	0.528 [0.393–0.657]	0.729
	2 and 3	41 (66.1%)					
	1 and 2	55 (88.7%)	9.5	80%	58.2%	0.545 [0.412–0.675]	0.735
	3	7 (11.3%)					
4	1	11 (17.7%)	10.7	31.2%	90.2%	0.588 [0.452–0.715]	0.341
	2 and 3	51 (82.3%)					
	1 and 2	49 (79%)	11.6	33.3%	97.9%	0.646 [0.511–0.766]	0.146
	3	13 (21%)					
5	1	6 (9.7%)	10.7	30.2%	100%	0.572 [0.437–0.700]	0.595
	2 and 3	56 (90.3%)					
	1 and 2	48 (77.4%)	10.54	61.5%	81.4%	0.713 [0.579–0.824]	0.018*
	3	14 (22.6%)					
6	1	7 (11.3%)	10.7	30.2%	100%	0.572 [0.437–0.700]	0.595
	2 and 3	55 (88.7%)					
	1 and 2	46 (74.2%)	9.08	57.1%	68.9%	0.543 [0.408–0.673]	0.654
	3	16 (25.8%)					
7	1	8 (12.9%)	10.67	31%	89.8%	0.517 [0.383–0.649]	0.899
	2 and 3	54 (87.1%)					
	1 and 2	36 (58.1%)	10.08	56.5%	70.6%	0.653 [0.515–0.774]	0.046*
	3	26 (41.9%)					

*AUC* area under curve, *95% CI* 95% confidence interval, *Statistically significant values

**Fig. 2:**
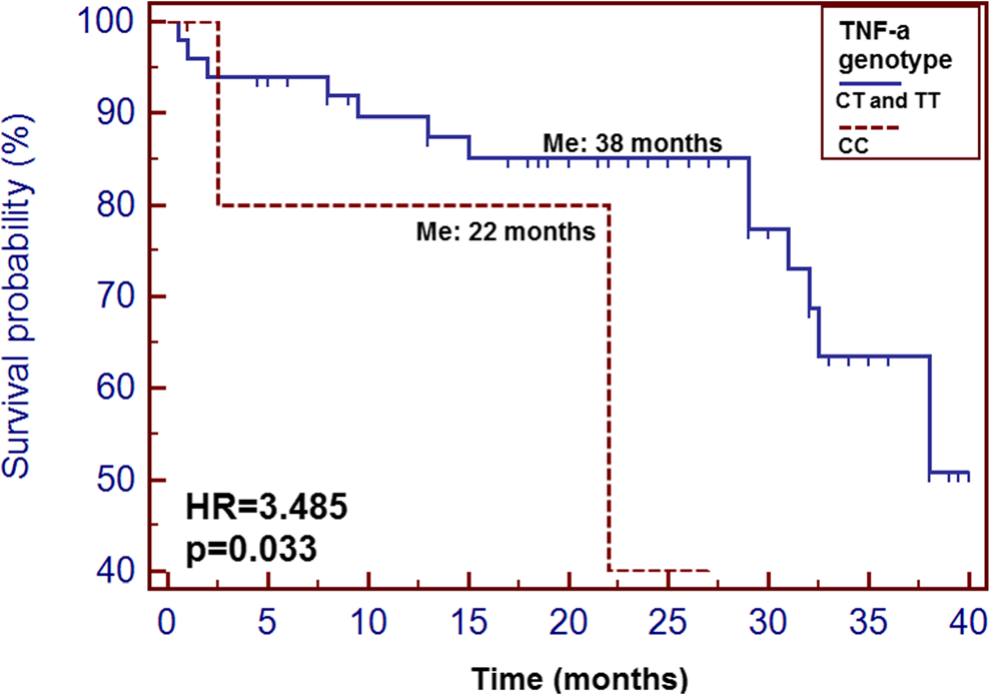
The probability of overall survival alteration depending on *TNF*-α genotype

Among classic prognostic factors, only histopathological diagnosis (other than squamous cell carcinoma) significantly increased risk of shorter OS (adjusted for other factors; 32.5 vs > 40 months; HR = 4.29, 95% CI 0.75–24.53; *p* = 0.048). Interestingly, high TNF-α level was significantly correlated with an increased risk of shorter OS (high vs low, 24 months vs 45 months; HR = 2.17, 95% CI 1.13–4.16; *p* = 0.0110; Fig. 2). Moreover, CC genotype carriers had significantly higher risk of shorter OS (HR = 6.84, 95% CI 1.24–37.73, *p* = 0.028) On the basis of Cox regression analysis (after adjustment for gender, age, histopathological diagnosis, stage of disease—TNM classification, performance status, alcohol consumption and tobacco smoking status, TNF-α plasma concentration and *TNF-α* genotype), we found that all three above (higher TNM stage, high TNF-α plasma concentration and CC genotype) were independent prognostic factors. The results of univariate and multivariate analysis are presented in Table 4.

**Table 4:** Overall survival analysis of HNC patients undergoing RTH depending on selected factors

Variable	Univariate		Multivariate
	Median (months)	*p* HR (95% CI)	*p* HR (95% CI)
Gender			
Women	32.5	0.555	0.500
Men	38	1.399 (0.404–4.872)	0.599 (0.136–2.634)
Age			
≥ 63	38	0.125	0.338
< 63	> 40	1.224 (0.429–3.494)	1.982 (0.493–7.959)
Histopathological diagnosis			
Plano	> 40	0.268	0.048*
Other	32.5	2.248 (0.284–17.772)	4.286 (0.749–24.530)
TNM stage			
I–III	> 40	0.750	0.990
IVA–IVC	36	0.817 (0.250–2.665)	0.996 (0.257–3.868)
Performance status			
1	> 40	0.765	0.955
2	38	0.737 (0.125–4.359)	0.939 (0.106–8.345)
Alcohol consumption			
Yes	> 40	0.768	0.475
No	38	1.159 (0.434–3.092)	0.638 (0.187–2.178)
Tobacco smoking			
Yes	38	0.219	0.282
No	> 40	3.300 (0.923–11.805)	3.338 (0.375–29.690)
TNF- α plasma concentration			
Low	> 40	0.354	0.444
High	32.5	1.570 (0.575–4.289)	1.504 (0.532–4.246)
*TNF- α* genotype			
CC	22	0.033*	0.028*
CT and TT	38	3.485 (0.458–26.54)	6.840 (1.240–37.730)

*HR* hazard ratio, *95% CI* 95% confidence interval, *Statistically significant values

## Discussion

In order to develop preventive and therapeutic options, it is necessary to comprehend the basic molecular mechanisms responsible for OM development. OM induced by RTH is an injury of the normal tissue. Since it occurs in 100% of altered fraction, RTH it is the main dose-limiting toxicity in HNC patients. This type of adverse effects is markedly related to maintaining the continuity of treatment and thus its efficiency (changes in the dose fractionation, poor local tumour control) and finally patient’s quality of life [4–7]. In clinical practice, we are not able to exactly define the risk of OM in HNC patients subjected to RTH; therefore, any dose escalation is always with the higher chance of healthy tissue damage. Classical risk factors for OM include C-RTH, poor oral hygiene, smoking, malnutrition or cachexia and lack of antibiotic use at the early stage of OM development [10]. However, even in the group of patients with the same characteristics, more severe OM develops only in some of them which suggests the important role of genetic predisposition [14]. The pathophysiology of OM is still not fully understood. It is suggested that OM develops in four main phases: an initial phase (based on inflammatory/vascular processes), an epithelial phase, an ulcerative/bacteriological phase (pseudomembranous) and a healing phase. In the inflammatory phase, local tissues (epithelial, connective and endovascular) injured by radiation release proinflammatory (e.g. TNF-α, interleukin 1-beta (IL-1β) and prostaglandins (PGs)) and anti-inflammatory (e.g. IL-10 and IL-11) cytokines. Change in the balance between these factors can lead both to increase of the tissue damage (by increased vascular permeability and thus higher intense recruitment and infiltration by inflammatory cells—in the case of pro-inflammatory cytokines) as well as to the limitation of the injury (anti-inflammatory cytokines). The epithelial phase depends mainly on epidermal growth factor (EGF) and keratinocyte growth factor (KGF) released to increase the rate of epithelial turnover after RTH-induced apoptosis. In the ulceration stage, loss of the protective barrier (disrupted basement membrane) results in microcoagulation and neutropenia which in turn facilitate secondary infections (due to gram-negative bacterias and yeast). Bacterial exotoxins exacerbate the inflammatory response leading to the release of more proinflammatory cytokines (including TNF-α and IL-1β) [15].

TNF-α serves as a ligand for TNFRSF1A (TNFR1) and TNFRSF1B (TNFR2) receptors. TNF-α - TNFR1 axis induce apoptosis whereas, signalling through the second pathway (TNFR2) promote the proliferation of tumour cells and suppressive immune cells [16]. Stimulation of oral cavity cells by overexpressed inflammatory cytokines including TNF-α (but also IL-1b, IL-6 and IL-8) seems to play a key role in OM development. The high concentration of TNF-α and other inflammatory molecules lead to decreased proliferation, migration as well as lower levels of growth factors important for maintaining oral mucosal homeostasis and appropriate dynamics of healing [17, 18]. The relationship between the intensity of OM and the level of TNF-α was assessed in several studies, however, hitherto obtained results were ambiguous. Some studies reported higher levels of TNF-α in irradiated patients, whereas others showed the opposite findings [19–22]. Only two studies demonstrated a significant correlation between TNF-α level and OM intensity. Haddad et al. showed, that patients with HNC subjected to RTH had significantly higher TNF-α plasma concentrations. Interestingly, they found a significant correlation between the increased concentration of this cytokine and more severe OM [23]. Similar significant association was noted by Xanthinaki et al. [20]. However, above findings were not confirmed by Meitovitz et al., because their study showed an inverse relationship (a decrease in TNF-α concentration in patients undergoing RTH) and a lack of association with OM severity [21]. On the other hand, more indirect evidence on the relationship between TNF-α levels and the response of cells to radiation and OM intensity was also provided by studies in animal models. In the above-mentioned studies, level of TNF-α decreased as a result of administration of benzydamine and IL-11, which led to modulation of tissue response to irradiation and thereby lower intense of OM [24–26]. Those inconclusive results can be partly explained by heterogeneous materials (used for TNF-α level evaluation: saliva, serum). Because existing studies assessing plasma TNF-α concentration did not give conclusive results, we suggest that genetic predispositions such as SNP of its gene (it may potentially affect both expression level and activity of encoded protein and is much more stable than the cytokine level—highly dynamic and multi-factor dependent, produced by both tumour tissues and by normal cells in reaction to different damaging factors) may be a better predictor of OM as well as prognostic factor in patients irradiated due to HNC. To date, many studies were trying to evaluate the correlation between genetic variants and radiosensitivity and the reaction of normal tissues to irradiation. However, most of the studies assessing the association of SNPs with OM are focused on the following processes: DNA repair, oxidation and stress response, apoptosis [14], embryogenesis [27], as well as inflammation [14], and only one (our previous paper) concerned TNF-α -TNFR axis [28].

In our previous study (58 HNC patients irradiated using IMRT technique), we observed that patients with T allele of *TNFRSF1A* gene (− 1187 T > G, rs4149570) demonstrated a higher risk of manifestation of grade 3 OM in the 5th, 6th and 7th week of RTH [28].

In a meta-analysis performed by Song et al. (17 studies: 656 patients and 2193 controls) a significant association between a wild type variant of *XRCC3* (722C > T, p.Thr241Met, rs861539) and acute reaction to irradiation among patients with various cancers was demonstrated. Moreover, in the subgroup of HNC patients, this SNP was also significantly associated with a higher risk of radiation adverse events [29].

One of the symptoms of RTH toxicity is the salivary secretion disorder caused by salivary glands exposure to high radiation doses. Reduced saliva production translates into the quality of life of the patient primarily arises dry mouth, difficulty swallowing and chewing, malnutrition, difficulty speaking and taste disorders. In addition to the dry mouth, the saliva formed is viscous; it tends to friction its physiological function and its composition is changed. Moreover, the lack of a physiological barrier that is saliva results in an increased risk of caries, bacterial and oral fungal infections or the occurrence of oral inflammation and mucositis. The toxic effect of RTH on the salivary glands is observed at doses above 52 Gy, and the reduction of salivary secretion usually occurs 1 week after the start of RTH [30–32]. Some studies suggest that salivary TNF-α may be a more useful marker for diagnostic and therapeutic purposes than the evaluation of this marker in serum [33]. In the saliva of patients treated with high doses of ionising radiation, the TNF-α level was higher than those exposed to lower values of radiation. In addition, the level of this marker evaluated in saliva significantly decreased after the end of treatment [34]. On the other hand, also in the case of the evaluation of TNF-α concentration in plasma samples from HNC patients subjected to IMRT, a significant decrease in the level of this cytokine after the end of treatment was found [35]. Above data suggests that plasma TNF-α may also serve as a useful predictor of RTH-related OM.

In our study group, OM was observed in all patients. Starting from weeks 2 and 3 of RTH, its intensity gradually increased. This is a typical reaction since first symptoms occur after application of 10–20 Gy, which usually takes 1–2 weeks. In terms of applied treatment, our study was relatively homogenous (in all patients IMRT technique was used for RTH). Moreover, all patients, receive similar: total doses: 60–66 Gy in adjuvant treatment and 70 Gy in alone radiotherapy, with fractioning of 2 Gy a day and irradiated tissue volumes (tumour or post-operative site and regional lymphatic nodes). All patients received the total planned dose and completed radiotherapy. 38.7% of patients were treated with C-RTH (1 to 4 courses of PF chemotherapy was administrated). C-RTH resulted in the significantly more frequent occurrence of 3 grade OM in weeks 5 and 7 of treatment. Interestingly, we observe a significant correlation between TNF-α protein level in serum and the grade OM in the 5th and 7th but not in the 6th week of observation. At the 6th week, the percentage of patients receiving concurrent C-RTH who developed OM 3-degree was lower (33.3%) than in 5th (41.7%) and 7th (66.7%) observation week. Patients received cytostatics (in concurrent C-RTH) in day 1 (week 1), 22 (week 3) and 43 (week 6) of treatment, this could have resulted in the more severe OM in 4th, 5th and 7th week of observation (1–2 weeks after the intensification of treatment). Patients with diabetes or collagen vascular disease were not included in the study. We did not observe any statistically significant differences in the occurrence and intensity of OM in relation to both age and gender. Other factors usually linked with higher intensity of OM (smoking, excessive alcohol use) had no statistical significance.

To our knowledge, this was the first study aimed at the evaluation of the correlation between SNP of TNF-α (− 1211 T > C) and plasma concentration of encoded by this gene cytokine and the occurrence and intensity of OM. Our study demonstrated that intensified OM after irradiation of the head and neck area can be found significantly more frequently in CC genotype carriers. However, naturally, our research has some limitations (sample size, different treatment regimens, TNF-α measurements performed only at a one-time point and assessment of generalised and not local inflammatory reaction—plasma instead of saliva as a tested material) and require further studies to confirm our results.

## Conclusions

HNC patients with CC genotype of the *TNF-α* gene (− 1211 T > C) have higher TNF-α plasma concentrations than those with T allele. Patients with the 3rd degree of OM have significantly higher TNF-α levels. The presence of CC genotype was related to a higher risk of 3rd degree OM development in the last weeks of RTH. The TNF-α level has good diagnostic accuracy in 3rd grade OM detection. Both TNF-α plasma concentration and SNP of TNF-α gene may modulate the risk of occurrence and intensity of OM in patients irradiated due to HNC. TNF-α may play a key role in the pathomechanism of development of OM in patients irradiated due to HNC. High TNF-α plasma concentration and CC genotype of TNF-α gene are independent prognostic factors for patients subjected to RTH due to HNC.

## Electronic supplementary material

ESM 1 (DOCX 36 kb)

## References

[1] Siegel RL, Miller KD, Jemal A (2016). Cancer statistics 2016. CA Cancer J Clin.

[2] Westra WH (2009). The changing face of head and neck cancer in the 21st century: the impact of HPV on the epidemiology and pathology of oral cancer (2099). Head Neck Pathol.

[3] Jaroneski LA (2006). The importance of assessment rating scales for chemotherapy-induced oral mucositis. Oncol Nurs Forum.

[4] Trotti A, Bellm LA, Epstein JB, Frame D, Fuchs HJ, Gwede CK, Komaroff E, Nalysnyk L, Zilberberg MD (2003). Mucositis incidence, severity and associated outcomes in patients with head and neck cancer receiving radiotherapy with or without chemotherapy: a systematic literature review. Radiother Oncol.

[5] Oronsky B, Goyal S, Kim MM, Cabrales P, Lybeck M, Caroen S, Oronsky N, Burbano E, Carter C, Oronsky A (2018). A review of clinical radioprotection and chemoprotection for oral mucositis. Transl Oncol.

[6] Minhas S, Kashif M, Altaf W, Afzal N, Nagi AH (2017). Concomitant-chemoradiotherapy-associated oral lesions in patients with oral squamous-cell carcinoma. Cancer Biol Med.

[7] Cox JD, Stetz J, Pajak TF (1995). Toxicity criteria of the radiation therapy oncology group (RTOG) and the European Organization for Research and Treatment of cancer (EORTC). Int J Radiat Oncol Biol Phys.

[8] Sonis ST, Elting LS, Keefe D, Peterson DE, Schubert M, Hauer-Jensen M, Bekele BN, Raber-Durlacher J, Donnelly JP, Rubenstein EB, for the Mucositis Study Section of the Multinational Association of Supportive Care in Cancer and the International Society for Oral Oncology (2004). Perspectives on cancer therapy-induced mucosal injury: pathogenesis, measurement, epidemiology, and consequences for patients. Cancer.

[9] Platek ME, McCloskey SA, Cruz M, Burke MS, Reid ME, Wilding GE (2013). Quantification of the effect of treatment duration on local-regional failure after definitive concurrent chemotherapy and intensity-modulated radiation therapy for squamous cell carcinoma of the head and neck. Head Neck.

[10] Vera-Llonch M, Oster G, Hagiwara M, Sonis S (2006). Oral mucositis in patients undergoing radiation treatment for head and neck carcinoma. Cancer.

[11] Normando AGC, Rocha CL, de Toledo IP, de Souza Figueiredo PT, Dos Reis PED, De Luca Canto G (2017). Biomarkers in the assessment of oral mucositis in head and neck cancer patients: a systematic review and meta-analysis. Support Care Cancer.

[12] Sonis ST (2002). The biologic role of nuclear factor-kB in disease and its potential involvement in mucosal injury associated with antineoplastic therapy. Crit Rev Oral Biol Med.

[13] Kalliolias GD, Ivashkiv LB (2016). TNF biology, pathogenic mechanisms and emerging therapeutic strategies. Nat Rev Rheumatol.

[14] Huang A, Glick SA (2017). Genetic susceptibility to cutaneous radiation injury. Arch Dermatol Res.

[15] Maria OM, Eliopoulos N, Muanza T (2017). Radiation-induced oral mucositis. Front Oncol.

[16] Sheng Y, Li F, Qin Z (2018). TNF receptor 2 makes tumor necrosis factor a friend of tumors. Front Immunol.

[17] Basso FG, Soares DG, Pansani TN, Cardoso LM, Scheffel DL, de Souza Costa CA, Hebling J (2016). Proliferation, migration, and expression of oral-mucosal-healing-related genes by oral fibroblasts receiving low-level laser therapy after inflammatory cytokines challenge. Lasers Surg Med.

[18] Akmansu M, Unsal D, Bora H, Elbeg S (2005). Influence of locoregional radiation treatment on tumor necrosis factor-alpha and interleukin-6 in the serum of patients with head and neck cancer. Cytokine.

[19] Citrin DE, Hitchcock YJ, Chung EJ, Frandsen J, Urick ME, Shield W, Gaffney D (2012). Determination of cytokine protein levels in oral secretions in patients undergoing radiotherapy for head and neck malignancies. Radiat Oncol.

[20] Xanthinaki A, Nicolatou-Galitis O, Athanassiadou P, Gonidi M, Kouloulias V, Sotiropoulou-Lontou A, Pissakas G, Kyprianou K, Kouvaris J, Patsouris E (2008). Apoptotic and inflammation markers in oral mucositis in head and neck cancer patients receiving radiotherapy: preliminary report. Support Care Cancer.

[21] Meirovitz A, Kuten M, Billan S, Abdah-Bortnyak R, Sharon A, Peretz T, Sela M, Schaffer M, Barak V (2010). Cytokines levels, severity of acute mucositis and the need of PEG tube installation during chemo-radiation for head and neck cancer - a prospective pilot study. Radiat Oncol.

[22] Seyyednejad F, Rezaee A, Haghi S, Goldust M (2012). Survey of pre-inflammation cytokines levels in radiotherapy-induced-mucositis. Pak J Biol Sci.

[23] Haddad R, Sonis S, Posner M, Wirth L, Costello R, Braschayko P, Allen A, Mahadevan A, Flynn J, Burke E, Li Y, Tishler RB (2009). Randomized phase 2 study of concomitant Chemoradiotherapy using weekly carboplatin/paclitaxel with or without daily subcutaneous Amifostine in patients with locally advanced head and neck cancer. Cancer.

[24] Keefe DM, Schubert MM, Elting LS, Sonis ST, Epstein JB, Raber-Durlacher JE, Migliorati CA, McGuire DB, Hutchins RD, Peterson DE, for the Mucositis Study Section of the Multinational Association of Supportive Care in Cancer, and the International Society for Oral Oncology (2007). Updated clinical practice guidelines for the prevention and treatment of mucositis. Cancer.

[25] Sonis ST, Watkins B, Fey E, Yuschak M, Parenti D (2005). Mechanism of action of benzydamine in the treatment of oral mucositis. J Clin Oncol.

[26] Sonis ST, Peterson RL, Edwards LJ, Lucey CA, Wang L, Mason L, Login G, Ymamkawa M, Moses G, Bouchard P, Hayes LL, Bedrosian C, Dorner AJ (2000). Defining mechanisms of action of interleukin-11 on the progression of radiation-induced oral mucositis in hamsters. Oral Oncol.

[27] Yu J, Huang Y, Liu L, Wang J, Yin J, Huang L, Chen S, Li J, Yuan H, Yang G, Liu W, Wang H, Pei Q, Guo C (2016). Genetic polymorphisms of Wnt/β-catenin pathway genes are associated with the efficacy and toxicities of radiotherapy in patients with nasopharyngeal carcinoma. Oncotarget.

[28] Brzozowska A, Powrózek T, Homa-Mlak I, Mlak R, Ciesielka M, Gołębiowski P, Małecka-Massalska T (2018). Polymorphism of promoter region of TNFRSF1A gene (−610 T > G) as a novel predictive factor for radiotherapy induced oral mucositis in HNC patients. Pathol Oncol Res.

[29] Song Y-Z, Han F-J, Liu M, Xia C-C, Shi W-Y, Dong L-H (2015). Association between single nucleotide polymorphisms in XRCC3 and radiation-induced adverse effects on normal tissue: a meta-analysis. PLoS One.

[30] Kornguth DG, Garden AS, Zheng Y, Dahlstrom KR, Wei Q, Sturgis EM (2005). Gastrostomy in oropharyngeal cancer patients with ERCC4 (XPF) germline variants. Int J Radiat Oncol Biol Phys.

[31] Likhterov I, Ru M, Ganz C, Urken ML, Chai R, Okay D, Liu J, Stewart R, Culliney B, Palacios D, Lazarus CL (2018). Objective and subjective hyposalivation after treatment for head and neck cancer: long-term outcomes. Laryngoscope.

[32] Nautiyal Vipul, Lal Punita, Verma Mranalini, Yadav Rajan, Maria Das KJ, Kumar Shaleen (2018). Objective and subjective assessment of xerostomia in patients of locally advanced head-and-neck cancers treated by intensity-modulated radiotherapy. Journal of Cancer Research and Therapeutics.

[33] Mozaffari HR, Ramezani M, Mahmoudiahmadabadi M, Omidpanah N, Sadeghi M (2017). Salivary and serum levels of tumor necrosis factor-alpha in oral lichen planus: a systematic review and meta-analysis study. Oral Surg Oral Med Oral Pathol Oral Radiol.

[34] Citrin DE, Hitchcock YJ, Chung EJ, Frandsen J, Urick ME, Shield W, Gaffney D (2012). Determination of cytokine protein levels in oral secretions in patients undergoing radiotherapy for head and neck malignancies. Radiat Oncol.

[35] Jin Y, Zhang G, Lin K, Chen X, Cui J, Wang Y (2017). Changes of plasma cytokines and chemokines expression level in nasopharyngeal carcinoma patients after treatment with definitive intensity-modulated radiotherapy (IMRT). PLoS One.

