# Supplementary material for: The relationship between TNF-α gene promoter polymorphism (− 1211 T > C), the plasma concentration of TNF-α, and risk of oral mucositis and shortening of overall survival in patients subjected to intensity-modulated radiation therapy due to head and neck cancer
Source: Support Care Cancer. 2019 May 10;28(2):531–40. doi: 10.1007/s00520-019-04838-6 (PMC6954128; doi:10.1007/s00520-019-04838-6)
Supplement: Supplementary file 1 — (DOCX 36 kb) [file 520_2019_4838_MOESM1_ESM.docx]

| ***TNF-α* genotype** | | | | | |
| --- | --- | --- | --- | --- | --- |
| **Factor** | | **CC**  **(n=6; 9.7%)** | **CT**  **(n=19; 30.6%)** | **TT**  **(n=37; 59.7%)** | ***p*** |
| **Gender** | **Male** | 3 (5.9%) | 16 (31.4%) | 32 (62.7%) | 0.100 |
|  | **Female** | 3 (27.3%) | 3 (27.3%) | 5 (45.4%) |  |
| **Age (years)** | **≥ 63** | 4 (12.5%) | 9 (28.1%) | 19 (59.4%) | 0.711 |
|  | **< 63** | 2 (6.7%) | 10 (33.3%) | 18 (60%) |  |
| **Performance status** | **<1** | 4 (7.3%) | 17 (30.9%) | 34 (61.8%) | 0.192 |
|  | **>1** | 2 (28.7%) | 2 (28.7%) | 3 (42.6%) |  |
| **Histopathological diagnosis** | **Squamous-cell carcinoma** | 6 (10.6%) | 17 (29.8%) | 34 (59.6%) | 0.711 |
|  | **Others** | 0 | 2 (40%) | 3 (60%) |  |
| **Diseasestage** | **I and III** | 2 (14.3%) | 5 (35.7%) | 7 (50%) | 0.115 |
|  | **IV** | 4 (8.3%) | 14 (29.2%) | 30 (62.5%) |  |
| **Tumor location** | **Upper throat** | 2 (11.8%) | 5 (29.4%) | 10 (58.8%) | 0.660 |
|  | **Lower throat** | 4 (8.9%) | 14 (31.1%) | 27 (60%) |  |
|  | **Larynx** | 3 (8.8%) | 12 (35.3%) | 19 (55.9%) | 0.111 |
|  | **Others** | 3 (10.7%) | 7 (25%) | 18 (64.3%) |  |
| **Alcohol consumption** | **Yes** | 4 (14.3%) | 9 (32.1%) | 15 (53.6%) | 0.372 |
|  | **No** | 2 (8.3%) | 10 (41.7%) | 12 (50%) |  |
| **Smoking status** | **Smoker** | 4 (7.7%) | 17 (32.7%) | 31 (59.6%) | 0.416 |
|  | **Non-smoker** | 2 (20%) | 2 (20%) | 6 (60%) |  |
|  | **Current smoker** | 3 (6.7%) | 14 (31.1%) | 28 (62.2%) | 0.579 |
|  | **Former smoker** | 1 (14.2%) | 3 (42.9%) | 3 (42.9%) |  |
| **Concurrent chemotherapy** | **Yes** | 3 (12.5%) | 8 (33.3%) | 13 (54.2%) | 0.736 |
|  | **No** | 3 (7.9%) | 11 (28.9%) | 24 (63.2%) |  |

**Supplementary table 1**. *TNF-α* genotype distribution according to patients’ clinical-demographic factors.

| ***TNF****-****α* gene SNP** | **TNF-α plasma concentration** | ***p*** |
| --- | --- | --- |
| **CC** (n=6; 9.7%) | 10.70±0.72 | 0.006* |
| **CT** (n=19; 30.6%) | 9.76±1.54 |  |
| **TT** (n=37; 59.7%) | 9.08±1.49 |  |
| **CC** (n=6; 9.7%) | 10.70±0.72 | 0.008* |
| **CT** **or TT** (n=56; 90.3%) | 9.62±1.39 |  |
| **CT** (n=19; 30.6%) | 9.76±1.54 | 0.002* |
| **CC** **or TT** (43; 69.4%) | 9.62±1.40 |  |
| **TT** (n=37; 59.7%) | 9.08±1.49 | 0.0015* |
| **CC or CT** (25; 40.3%) | 9.98±1.37 |  |

**Supplementary table 2**.TNF-α level depending on *TNF-a* gene genotype variant.

*statistically significant values.

| **Factor** | | **RTH cycle and grade of radiation induced oral mucositis** | | | | | | | | |
| --- | --- | --- | --- | --- | --- | --- | --- | --- | --- | --- |
|  |  | **5th cycle** | | ***p*,**  **OR [95%CI]** | **6th cycle** | | ***p*,**  **OR [95%CI]** | **7th cycle** | | ***p*,**  **OR [95%CI]** |
|  |  | **1** | **2 and 3** |  | **1** | **2 and 3** |  | **1** | **2 and 3** |  |
| **Gender** | **Male** | 6 (11.8%) | 45 (88.2%) | 0.429  3.286 [0.172-62.673] | 6 (11.8%) | 45 (88.2%) | 0.800  1.333 [0.144-12.340] | 5 (9.8%) | 46 (90.2%) | 0.133  3.450 [0.685-17.370] |
|  | **Female** | - | 11 (100%) |  | 1 (9.1%) | 10 (90.9%) |  | 3 (27.3%) | 8 (72.7%) |  |
| **Age** | **≥63** | 5 (15.6%) | 27 (84.4%) | 0.136  5.370 [0.589-48.961] | 5 (15.6%) | 27 (84.4%) | 0.279  2.593 [0.463-14.521] | 5 (15.6%) | 27 (84.4%) | 0.512  1.667 [0.362-7.678] |
|  | **<63** | 1 (3.3%) | 29 (96.7%) |  | 2 (6.7%) | 28 (93.3%) |  | 3 (10%) | 27 (90%) |  |
| **Disease**  **stage** | **I and III** | 2 (14.3%) | 12 (85.7%) | 0.512  1.833 [0.299-11.241] | 2 (14.3%) | 12 (85.7%) | 0.512  1.833 [0.299-11.241] | 2 (14.3%) | 12 (85.7%) | 0.512  1.833 [0.299-11.241] |
|  | **IV** | 4 (8.3%) | 44 (91.7%) |  | 4 (8.3%) | 44 (91.7%) |  | 4 (8.3%) | 44 (91.7%) |  |
| **Smoking history** | **Smoker** | 4 (7.7%) | 48 (92.3%) | 0.246  3.000 [0.469-19.177] | 5 (9.6%) | 47 (90.4%) | 0.353  2.350 [0.387-14.260] | 7 (13.5%) | 45 (86.5%) | 0.766  1.400 [0.153-12.816] |
|  | **Never smoker** | 2 (20%) | 8 (80%) |  | 2 (20%) | 8 (80%) |  | 1 (10%) | 9 (90%) |  |
| **Alcohol consumption** | **Yes** | 2 (7.1%) | 26 (92.9%) | 0.544  1.733 [0.293-10.246] | 2 (7.1%) | 26 (92.9%) | 0.359  2.241 [0.400-12.557] | 1 (3.6%) | 27 (96.4%) | 0.078  7.00 [0.806-60.830] |
|  | **No** | 4 (11.8%) | 30 (88.2%) |  | 5 (14.7%) | 29 (85.3%) |  | 7 (20.6%) | 27 (79.4%) |  |
| **Concurent chemotherapy** | **Yes** | - | 24 (100%) | 0.126  9.800 [0.527-182.42] | - | 24 (100%) | 0.100  11.67 [0.635-214.39] | - | 24 (100%) | 0.077  13.66 [0.750-248.52] |
|  | **No** | 6 (15.8%) | 32 (84.2%) |  | 7 (18.4%) | 31 (81.6%) |  | 8 (21%) | 30 (79%) |  |
| **Neoadjuvant**  **chemotherapy** | **Yes** | - | 10 (100%) | 0.475  2.936 [0.153-56.277] | - | 10 (100%) | 0.475  2.936 [0.153-56.277] | - | 10 (100%) | 0.475  2.936 [0.153-56.277] |
|  | **No** | 6 (11.5%) | 46 (88.5%) |  | 6 (11.5%) | 46 (88.5%) |  | 6 (11.5%) | 46 (88.5%) |  |

**Supplementary table 3**. Impact of demographic and clinical factors on the risk of moderate or severe oral mucositis after subsequent cycles of RTH.

OR - odds ratio, 95% CI - 95% confidence interval.

| **Factor** | | **RTH cycle and grade of radiation-induced oral mucositis** | | | | | | | | |
| --- | --- | --- | --- | --- | --- | --- | --- | --- | --- | --- |
|  |  | **5th cycle** | | ***p*,**  **OR [95%CI]** | **6th cycle** | | ***p*,**  **OR [95%CI]** | **7th cycle** | | ***p*,**  **OR [95%CI]** |
|  |  | **1 and 2** | **3** |  | **1 and 2** | **3** |  | **1 and 2** | **3** |  |
| **Gender** | **Male** | 41 (80.4%) | 10 (19.6%) | 0.237  2.343 [0.572-1.522] | 40 (78.4%) | 11 (21.6%) | 0.111  3.030 [0.777-11.825] | 30 (58.8%) | 21 (41.2%) | 0.794  1.191 [0.321-4.418] |
|  | **Female** | 7 (63.6%) | 4 (36.4%) |  | 6 (54.5%) | 5 (45.5%) |  | 6 (54.5%) | 5 (45.5%) |  |
| **Age** | **≥63** | 27 (84.4%) | 5 (15.6%) | 0.182  2.314 [0.674-7.942] | 26 (81.2%) | 6 (18.8%) | 0.195  2.167 [0.674-6.967] | 22 (68.7%) | 10 (31.3%) | 0.081  2.514 [0.892-7.085] |
|  | **<63** | 21 (70%) | 9 (30%) |  | 20 (66.7%) | 10 (33.3%) |  | 14 (46.7%) | 16 (53.3%) |  |
| **Disease**  **stage** | **I and III** | 11 (78.6%) | 3 (21.4%) | 0.907  1.090 [0.257-4.616] | 9 (64.3%) | 5 (35.7%) | 0.340  1.869 [0.518-6.747] | 9 (64.3%) | 5 (35.7%) | 0.593  1.400 [0.408-4.804] |
|  | **IV** | 37 (77.1%) | 11 (22.9%) |  | 37 (77.1%) | 11 (22.9%) |  | 27 (56.2%) | 21 (43.8%) |  |
| **Smoking history** | **Smoker** | 41 (78.8%) | 11 (21.2%) | 0.543  1.597 [0.354-7.212] | 39 (75%) | 13 (25%) | 0.741  1.286 [0.289-5.711] | 31 (59.6%) | 21 (40.4%) | 0.574  1.476 [0.380-5.739] |
|  | **Neve-smoker** | 7 (70%) | 3 (30%) |  | 7 (70%) | 3 (30%) |  | 5 (50%) | 5 (50%) |  |
| **Alcohol consumption** | **Yes** | 21 (75%) | 7 (25%) | 0.680  1.286 [0.390-4.238] | 19 (67.9%) | 9 (32.1%) | 0.304  1.827 [0.579-5.764] | 14 (50%) | 14 (50%) | 0.245  1.833 [0.660-5.092] |
|  | **No** | 27 (79.4%) | 7 (20.6%) |  | 27 (79.4%) | 7 (20.6%) |  | 22 (64.7%) | 12 (35.3%) |  |
| **Concurent chemotherapy** | **Yes** | 14 (58.3%) | 10 (41.7%) | 0.007*  6.071 [1.628-22.638] | 16 (66.7%) | 8 (33.3%) | 0.285  1.875 [0.592-5.937] | 8 (33.3%) | 16 (66.7%) | 0.002*  5.600 [1.838-17.066] |
|  | **No** | 34 (89.5%) | 4 (10.5%) |  | 30 (79%) | 8 (21%) |  | 28 (73.7%) | 10 (26.3%) |  |
| **Neoadjuvant**  **chemotherapy** | **Yes** | 7 (70%) | 3 (30%) | 0.543  1.597 [0.354-7.212] | 6 (60%) | 4 (40%) | 0.270  2.222 [0.537-9.195] | 4 (40%) | 6 (60%) | 0.215  2.400 [0.602-9.569] |
|  | **No** | 41 (78.8%) | 11 (21.2%) |  | 40 (76.9%) | 12 (23.1%) |  | 32 (61.5%) | 20 (38.5%) |  |

**Supplementary table 4**. Impact of demographic and clinical factors on the risk of severe oral mucositis after subsequent cycles of RTH.

OR - odds ratio, 95% CI - 95% confidence interval, *statistically significant values.

| **RTH cycle** | **Radiation reaction grade** | **CC (n=6; 9.7%)** | **CT (n=19; 30.6%)** | **TT (n=37; 59.7%)** | ***p*** | **TNF**-**α concentration** | ***p*** |
| --- | --- | --- | --- | --- | --- | --- | --- |
| **1** | **0**(n=6; 9.7%) | 0 | 1 (16.7%) | 5 (83.3%) | 0.430 | 9.62±1.39 | ns |
|  | **1**(n=56; 90.3%) | 6 (10.8%) | 18 (32.1%) | 32 (57.1%) |  | 9.64±1.38 |  |
| **2** | **1**(n=36; 58%) | 2 (5.5%) | 11 (30.6%) | 23 (63.9%) | 0.414 | 9.94±1.51 | 0.639 |
|  | **2**(n=26; 42%) | 4 (15.4%) | 8 (30.8%) | 14 (53.8%) |  | 9.83±1.53 |  |
| **3** | **1**(n=21; 33.9%) | 1 (4.7%) | 6 (28.6%) | 14 (66.7%) | 0.578 | 9.92±1.56 | 0.727 |
|  | **2 and 3**(n=41; 66.1%) | 5 (12.2%) | 13 (31.7%) | 23 (56.1%) |  | 9.88±1.50 |  |
|  | **1 and 2**(n=55; 88.7%) | 5 (9.1%) | 15 (27.3%) | 35 (63.6%) | 0.196 | 9.91±1.53 | 0.643 |
|  | **3**(n=7; 11.3%) | 1 (14.3%) | 4 (57.1%) | 2 (28.6%) |  | 9.73±1.53 |  |
| **4** | **1**(n=11; 17.7%) | 0 | 2 (18.2%) | 9 (81.8%) | 0.215 | 9.43±1.54 | 0.365 |
|  | **2 and 3**(n=51; 82.3%) | 6 (11.8%) | 17 (33.3%) | 28 (54.9%) |  | 10.0±1.53 |  |
|  | **1 and 2**(n=49; 79%) | 4 (8.2%) | 13 (26.5%) | 32 (65.3%) | 0.214 | 9.73±1.51 | 0.152 |
|  | **3**(n=13; 21%) | 2 (15.3%) | 6 (46.2%) | 5 (38.5%) |  | 10.55±1.53 |  |
| **5** | **1**(n=6; 9.7%) | 0 | 0 | 6 | 0.106 | 9.33±1.62 | 0.563 |
|  | **2 and 3**(n=56; 90.3%) | 6 (10.7%) | 19 (33.9%) | 31 (55.4%) |  | 9.96±1.53 |  |
|  | **1 and 2** (n=48; 77.4%) | 4 (8.3%) | 13 (27.1%) | 31 (64.6%) | 0.344 | 9.45±1.45 | 0.020* |
|  | **3** (n=14; 22.6%) | 2 (14.2%) | 6 (42.9%) | 6 (42.9%) |  | 10.40±1.55 |  |
| **6** | **1** (n=7; 11.3%) | 0 | 0 | 7 | 0.07 | 9.33±1.62 | 0.563 |
|  | **2 and 3** (n=55; 88.7%) | 6 (11%) | 19 (34.5%) | 30 (54.5%) |  | 9.96±1.54 |  |
|  | **1 and 2** (n=46; 74.2%) | 2 (4.3%) | 16 (34.8%) | 28 (60.9%) | 0.044* | 9.75±1.54 | 0.630 |
|  | **3**(n=16; 25.8%) | 4 (25%) | 3 (18.7%) | 9 (56.3%) |  | 9.70±1.50 |  |
| **7** | **1**(n=8; 12.9%) | 0 | 0 | 8 | 0.045* | 9.5±1.41 | 0.883 |
|  | **2 and 3**(n=54; 87.1%) | 6 (11.1%) | 19 (35.2%) | 29 (53.7%) |  | 9.91±1.60 |  |
|  | **1 and 2**(n=36; 58.1%) | 0 | 8 (22.2%) | 28 (77.8%) | <0.001* | 9.60±1.59 | 0.043* |
|  | **3**(n=26; 41.9%) | 6 (23.1%) | 11 (42.3%) | 9 (34.6%) |  | 10.32±1.60 |  |

**Supplementary table 5**.*TNF-α* genotype distribution according to patients' radiation-induced oral mucositis severity after subsequent cycles of RTH.

*statistically significant values.
